# Tobacco smoking by disability status before and after COVID-19 onset: a repeated cross-sectional analysis of 1 087 678 adults

**DOI:** 10.1093/eurpub/ckaf214

**Published:** 2025-11-22

**Authors:** Yusuff Adebayo Adebisi, Najim Z Alshahrani, Oshibe Joseph Daberechi, Isaac Olushola Ogunkola, Don Eliseo Lucero-Prisno

**Affiliations:** College of Social Sciences, University of Glasgow, Glasgow, United Kingdom; Department of Family and Community Medicine, Faculty of Medicine, University of Jeddah, Jeddah, Saudi Arabia; Medical Laboratory Science Department, Ebonyi State University, Abakaliki, Nigeria; Nuffield Department of Population Health, University of Oxford, Oxford, United Kingdom; Bristol Medical School, University of Bristol, Bristol, United Kingdom; Department of Global Health and Development, London School of Hygiene and Tropical Medicine, London, United Kingdom; Center for University Research, University of Makati, Makati City, Philippines; Research Office, Palompon Institute of Technology, Palompon, Leyte, Philippines

## Abstract

Smoking remains a leading cause of preventable illness and death in the United Kingdom, but little is known about recent trends in smoking disparities between disabled and non-disabled adults, particularly in the context of the coronavirus disease 2019 (COVID-19) pandemic. We analysed UK Annual Population Survey data from 2017 to 2023 for adults aged ≥18 years. Smoking status was classified as current, ex-, or never smoker, and disability status was defined according to the Equality Act. Multinomial logistic regression was used to estimate pooled adjusted relative risk ratios (RRRs) for smoking outcomes by disability status, and then separately for periods before (2017–19) and after (2020–23) the pandemic onset. To test whether the pandemic had an effect beyond underlying trends, we fitted a joint model including survey year and a post-2020 indicator. Adjusted average marginal effects quantified absolute percentage point (pp) differences in predicted probabilities between disabled and non-disabled adults. The analytic sample comprised 1 087 678 adults, of whom 26.7% (*n* = 290 536) reported a disability. In pooled adjusted analyses covering all survey years, and controlling for age, sex, education, ethnicity, marital status, and region of residence, disabled adults had higher relative risk ratios of being current smokers (RRR = 1.78; 95% CI: 1.75–1.80; *P < .*001) and ex-smokers (RRR = 1.44; 95% CI: 1.42–1.45; *P < .*001) compared with never smokers. Period-stratified analyses (not adjusted for temporal trends) showed adjusted RRRs for current smoking of 1.65 (95% CI: 1.62–1.68; *P < .*001) before and 1.95 (95% CI: 1.91–1.99; *P < .*001) after the pandemic onset. In the fully adjusted joint model accounting for temporal trends (survey year) and pandemic period, the disability × pandemic period interaction was not statistically significant (χ^2^ = 3.11; *P = .*21). Adjusted average marginal effects from the trend-adjusted model showed that disabled adults had a higher predicted probability of current smoking both before (+5.63 percentage points; 95% CI: 5.30–5.96; *P < .*001) and after (+4.60 pp; 95% CI: 4.25–4.96; *P < .*001) the pandemic onset, representing a modest narrowing of the absolute gap (difference = −1.03 pp; *P = .*001). Disabled adults remained substantially more likely to smoke than their non-disabled counterparts. After accounting for underlying temporal trends, the onset of the COVID-19 pandemic did not independently change this association, highlighting the continued need for disability-inclusive cessation strategies.

## Introduction

Tobacco smoking is a leading cause of preventable illness and death in the United Kingdom, responsible for around 76 000 deaths each year and imposing significant healthcare and economic costs [1]. Smoking is a major driver of health inequalities, with disadvantaged groups bearing a disproportionate share of tobacco-related illness [2–4]. Although smoking prevalence has fallen sharply over recent decades, from more than half of men and two-fifths of women in the 1970s to 11.9% of adults in 2023, these national averages mask deep and persistent disparities across socioeconomic, regional, and demographic lines [5]. People with disabilities represent one such group, with multiple studies reporting that they have higher smoking rates, greater nicotine dependence, and reduced success in quitting compared with the general population [6–8]. These disparities contribute to the overall poorer health outcomes experienced by disabled people, reinforcing cycles of disadvantage.

Under the Equality Act 2010, disability is defined as a physical or mental impairment that has a substantial and long-term adverse effect on an individual’s ability to carry out normal day-to-day activities [9]. This legal definition captures a diverse set of conditions, from mobility limitations and sensory impairments to chronic illnesses and mental health disorders [10]. Disabled adults are more likely to experience a range of social and economic disadvantages that can increase smoking risk, including lower household income, unemployment or economic inactivity, reduced educational opportunities, and greater exposure to stressors such as social isolation and discrimination [11, 12]. Many live with multiple long-term health conditions, which may both increase the perceived benefits of smoking for coping with pain or distress and reduce opportunities to access cessation support [6]. Barriers to quitting can include lack of accessible services, insufficient tailoring of cessation interventions to complex health needs, and stigma in healthcare settings, which may undermine trust and willingness to seek help [13–15]. Together, these factors create a context in which smoking can become both more prevalent and more entrenched.

Despite the recognized association between disability and smoking, routine national monitoring in the UK often fails to provide detailed breakdowns of tobacco use by disability status [5]. Large-scale surveillance reports frequently present aggregated figures for the general population or focus on other forms of inequality, such as those related to income, education, or region. Peer-reviewed research exploring disability-specific smoking patterns is limited, and even fewer studies have examined how these disparities have changed over time. This lack of granular evidence makes it difficult to assess whether tobacco control policies are benefiting disabled adults to the same extent as other groups. Addressing this knowledge gap is critical because without regular, disaggregated data, public health strategies may inadvertently perpetuate or widen inequalities in smoking prevalence. This issue has gained further urgency given that disabled people often face intersecting disadvantages, meaning that even modest gaps in prevalence can translate into substantial differences in health outcomes over time.

The coronavirus disease 2019 (COVID-19) pandemic has amplified the importance of understanding how smoking behaviours vary between disabled and non-disabled populations. From early 2020, the UK experienced widespread social, economic, and health system disruption, including lockdowns, shielding requirements for clinically vulnerable groups, reduced access to face-to-face healthcare, and changes in income and employment patterns [16]. Disabled adults were disproportionately affected by these measures due to higher representation among those advised to shield, greater dependence on disrupted support services, and increased susceptibility to severe illness from COVID-19 [17]. International evidence suggests that pandemic-related stress, social isolation, and heightened health anxieties produced divergent smoking responses, with some individuals increasing tobacco use and others reducing or quitting [18]. However, vulnerable groups often experienced the least favourable shifts, potentially due to limited access to cessation support during periods when public health resources were redirected. In the UK, national surveys indicate that smoking prevalence continued to decline modestly during the pandemic [19], but there is insufficient evidence on whether these declines were shared equally across disability status. Understanding this dynamic is vital to ensuring that future tobacco control measures are inclusive and equitable.

This study addresses these gaps by analysing pooled, nationally representative cross-sectional data from UK adults surveyed between 2017 and 2023. We estimate the association between disability status and smoking status (current smoker, ex-smoker, never smoked), assess whether these associations changed after the onset of the COVID-19 pandemic, and quantify absolute differences in predicted probabilities before and after 2020. By examining both relative and absolute measures, and by accounting for underlying year-on-year trends in a joint model including survey year and a post-2020 indicator, we provide new evidence on the persistence and evolution of smoking disparities between disabled and non-disabled adults in the UK.

## Methods

### Study design, data source, and participants

We conducted a repeated cross-sectional analysis using individual-level data from the UK Annual Population Survey (APS) for the period January 2017 to December 2023. The APS is a large, continuous household survey conducted by the Office for National Statistics (ONS) that combines results from the Labour Force Survey and local area boost samples to provide representative estimates for the UK population [20]. The survey covers adults aged 16 years and over living in private households in England, Wales, Scotland, and Northern Ireland, and collects detailed information on sociodemographic characteristics, employment, health, and lifestyle behaviours, including smoking status [20].

The initial dataset comprised 1 620 133 respondents across the 7 survey years: 2017 (*n* = 290 060), 2018 (*n* = 284 104), 2019 (*n* = 277 115), 2020 (*n* = 217 194), 2021 (*n* = 212 976), 2022 (*n* = 192 265), and 2023 (*n* = 146 419). For the purposes of this analysis, we restricted the sample to respondents aged 18 years and over with valid information on smoking status and disability. We excluded individuals aged under 18 years (*n* = 322 325), those with missing or non-response codes for disability (*n* = 162 933), and those with missing or non-response codes for smoking status (*n* = 47 197). After exclusions, the final analytic sample comprised 1 087 678 adults.

### Study variables

The primary exposure was disability status, defined using the Equality Act definition included in the APS [9]. Respondents were classified as ‘disabled’ if they reported having a physical or mental health condition or illness lasting 12 months or more that reduced their ability to carry out day-to-day activities [9]. All other respondents were classified as ‘not disabled’. Individuals coded as ‘does not apply’ or ‘no answer’ were excluded from the analysis (*n* = 162 933).

The main outcome was self-reported smoking status, derived from the harmonized APS variable CIGSMK16. Respondents were classified into three mutually exclusive categories: current cigarette smoker, ex-smoker, and never smoked. Responses coded as ‘does not apply’ or ‘no answer’ were excluded (*n* = 47 197).

The covariates were selected *a priori* based on existing literature and theoretical relevance as potential confounders of the relationship between disability and smoking [6–8]. These included age (grouped into 18–24, 25–34, 35–49, 50–64, and 65 years or older), sex (male or female), highest educational attainment (degree or equivalent, higher education below degree level, GCE A-level equivalent, GCSE A*–C equivalent, other qualification, or no qualification, with a separate category for missing or inapplicable responses), ethnicity (White, Mixed/Multiple ethnic groups, Asian, Black, Other ethnic group, or unknown/no answer), marital status (single/never married, married or living with a spouse, separated, divorced, widowed, or in a civil partnership), and region (based on the 13-category APS classification, which lists Merseyside separately from the North West, plus separate categories for Wales, Scotland, and Northern Ireland).

To classify periods relative to the COVID-19 pandemic, we defined ‘before onset’ as survey years 2017–19 and ‘after onset’ as 2020–23. This definition was chosen to reflect the introduction of national restrictions in March 2020 and subsequent social and economic changes [21].

### Statistical analyses

We first generated descriptive statistics to characterize the study population. Counts and percentages were calculated for smoking status, stratified by disability status and by period relative to the onset of the COVID-19 pandemic.

Because the outcome comprised more than two unordered categories, we used multinomial logistic regression to estimate the association between disability status and smoking status. This method enables simultaneous estimation of relative risk ratios for being a current smoker or an ex-smoker compared with never having smoked, while accounting for the nominal nature of the dependent variable. Model adequacy was assessed by testing the independence of irrelevant alternatives (IIA) assumption using the Small–Hsiao test, which supported the appropriateness of the multinomial specification (*P* > .05).

We estimated a crude multinomial logistic regression model including only disability status as the independent variable. Subsequently, adjusted models were fitted controlling for covariates selected *a priori* based on existing literature and theoretical relevance as potential confounders: age group, sex, highest educational attainment, ethnicity, marital status, and government office region. To assess whether associations differed before and after the onset of the COVID-19 pandemic, separate adjusted models were estimated for the pre-pandemic (2017–19) and post-pandemic (2020–23) periods. Relative risk ratios (RRRs) and corresponding 95% confidence intervals (CIs) were reported for all models. These stratified analyses were unadjusted for survey year and therefore reflect period differences without accounting for underlying temporal trends.

To determine whether any observed change reflected a true pandemic effect or continuation of pre-existing trends, we fitted a joint ‘trend-adjusted’ segmented regression model including both survey year (continuous, centred at 2019) and a post-2020 indicator, along with disability × year and disability × period interaction terms. This specification tested whether disability-related differences in smoking persisted or changed once underlying year-on-year trends were accounted for. Adjusted RRRs and adjusted average marginal effects (AMEs) were derived, and a Wald test assessed the joint significance of the disability × pandemic period and disability × survey year interactions. Pairwise contrasts of AMEs estimated the change in the disability gap in smoking status before and after 2020, with corresponding 95% CIs and *P* values.

Statistical significance was set at *P < .*05. All analyses were conducted using Stata MP version 18.0 (StataCorp LLC, College Station, TX, USA).

## Results

### Sample characteristics

Table 1 summarizes the characteristics of 1 087 678 UK adults surveyed between 2017 and 2023, stratified by disability status. Disabled adults (*n* = 290 536) were markedly older than their non-disabled counterparts, with 69.3% aged 50 years or older compared with 48.1% among non-disabled adults. Women comprised a greater proportion of the disabled group (56.9% vs. 50.9%). The majority in both groups were White, though the proportion was slightly higher among disabled adults (93.1% vs. 89.8%). Disabled adults had lower educational attainment; for example, only 11.6% held a degree compared with 24.4% of non-disabled adults, and 9.8% had no qualifications versus 4.6% of non-disabled adults. They were also less likely to be married or living with a spouse (48.4% vs. 55.7%) and more likely to be widowed (10.5% vs. 3.8%) or divorced (12.2% vs. 7.4%). Regional distributions were broadly similar, though slightly higher proportions of disabled adults lived in Wales, Scotland, and the North East of England. Smoking status differed substantially: 17.5% of disabled adults were current smokers compared with 11.7% of non-disabled adults, while fewer disabled adults had never smoked (49.2% vs. 64.0%).

**Table 1.** Sample characteristics by disability status, UK adults (pooled 2017–23)^a^

Characteristic	Disabled (*n* = 290 536)	Not disabled (*n* = 797 142)	Total (*n* = 1 087 678)
Age group			
18–24	13 102 (4.5)	70 985 (8.9)	84 087 (7.7)
25–34	24 510 (8.4)	123 980 (15.6)	148 490 (13.6)
35–49	51 471 (17.7)	218 619 (27.4)	270 090 (24.8)
50–64	93 059 (32.0)	239 816 (30.1)	332 875 (30.6)
65+	108 394 (37.3)	143 742 (18.0)	252 136 (23.2)
Sex			
Male	125 193 (43.1)	391 174 (49.1)	516 367 (47.5)
Female	165 343 (56.9)	405 968 (50.9)	571 311 (52.5)
Ethnicity			
White	270 440 (93.1)	715 826 (89.8)	986 266 (90.7)
Mixed/multiple	2122 (0.7)	6937 (0.9)	9059 (0.8)
Asian	10 997 (3.8)	46 051 (5.8)	57 048 (5.2)
Black	4226 (1.5)	17 619 (2.2)	21 845 (2.0)
Other ethnic group	2616 (0.9)	10 315 (1.3)	12 931 (1.2)
Unknown/no answer	135 (0.05)	394 (0.05)	529 (0.05)
Highest education			
Degree or equivalent	33 718 (11.6)	194 824 (24.4)	228 542 (21.0)
Higher education	15 096 (5.2)	55 350 (6.9)	70 446 (6.5)
GCE A level equivalent	33 610 (11.6)	129 503 (16.3)	163 113 (15.0)
GCSE A*–C equivalent	36 457 (12.6)	108 463 (13.6)	144 920 (13.3)
Other qualification	16 127 (5.6)	41 997 (5.3)	58 124 (5.3)
No qualification	28 380 (9.8)	36 818 (4.6)	65 198 (6.0)
Unknown/not applicable	127 148 (43.8)	230 187 (28.9)	357 335 (32.9)
Marital status			
Single, never married	74 314 (25.6)	244 230 (30.6)	318 544 (29.3)
Married/living with spouse	140 626 (48.4)	443 993 (55.7)	584 619 (53.8)
Separated	8731 (3.0)	16 548 (2.1)	25 279 (2.3)
Divorced	35 545 (12.2)	59 257 (7.4)	94 802 (8.7)
Widowed	30 481 (10.5)	30 618 (3.8)	61 099 (5.6)
Civil partnership	839 (0.3)	2496 (0.3)	3335 (0.3)
Region			
North East	20 759 (7.2)	47 502 (6.0)	68 261 (6.3)
North West	27 488 (9.5)	71 296 (8.9)	98 784 (9.1)
Merseyside	7383 (2.5)	17 739 (2.2)	25 122 (2.3)
Yorkshire & Humberside	25 069 (8.6)	61 485 (7.7)	86 554 (8.0)
East Midlands	16 317 (5.6)	41 913 (5.3)	58 230 (5.3)
West Midlands	22 942 (7.9)	62 261 (7.8)	85 203 (7.8)
Eastern	19 321 (6.7)	57 784 (7.3)	77 105 (7.1)
London	17 054 (5.9)	63 980 (8.0)	81 034 (7.5)
South East	30 100 (10.4)	97 174 (12.2)	127 274 (11.7)
South West	25 554 (8.8)	69 532 (8.7)	95 086 (8.7)
Wales	31 965 (11.0)	79 254 (9.9)	111 219 (10.2)
Scotland	33 647 (11.6)	93 498 (11.7)	127 145 (11.7)
Northern Ireland	12 937 (4.5)	33 724 (4.2)	46 661 (4.3)
Survey year			
2017	47 401 (16.3)	145 941 (18.3)	193 342 (17.8)
2018	47 889 (16.5)	140 446 (17.6)	188 335 (17.3)
2019	48 186 (16.6)	134 692 (16.9)	182 878 (16.8)
2020	37 208 (12.8)	107 090 (13.4)	144 298 (13.3)
2021	40 482 (13.9)	106 952 (13.4)	147 434 (13.5)
2022	39 026 (13.4)	92 615 (11.6)	131 641 (12.1)
2023	30 344 (10.4)	69 406 (8.7)	99 750 (9.2)
Smoking status			
Current smoker	50 722 (17.5)	93 015 (11.7)	143 737 (13.2)
Ex-smoker	96 969 (33.4)	194 282 (24.3)	291 251 (26.8)
Never smoked	142 845 (49.1)	509 845 (64.0)	652 690 (60.0)

a Percentages are unweighted and columns sum to 100%. ‘Unknown/not applicable’ refers primarily to respondents for whom education data were not collected in the 2022–23 APS waves or were obtained via proxy responses. These cases reflect survey design rather than missing data.

### Cross-sectional smoking prevalence before and after COVID-19 onset

Table 2 presents smoking status by disability status and period relative to the onset of the COVID-19 pandemic. Before the pandemic (2017–19), current smoking prevalence was substantially higher among disabled adults (20.0%) than non-disabled adults (13.7%). After the pandemic began (2020–23), current smoking declined in both groups, but the relative difference persisted, with 15.0% of disabled adults and 9.4% of non-disabled adults smoking. Ex-smoking was also more common among disabled adults, increasing from 32.5% before the pandemic to 34.3% after, compared with 23.9% to 24.9% among non-disabled adults. Conversely, never-smoking was markedly less common in the disabled group, though it rose slightly after the pandemic (47.5%–50.8%), remaining well below the prevalence among non-disabled adults (62.3%–65.8%). Differences in smoking status distributions by disability and period were statistically significant (Pearson χ^2^(6) = 25 000, *P < .*001).

**Table 2.** Smoking status by disability status and period relative to COVID-19 pandemic onset (pooled 2017–23)^a^

Smoking status	Not disabled—before onset (2017–19)	Not disabled—after onset (2020–23)	Disabled—before onset (2017–19)	Disabled—after onset (2020–23)	Total
Current cigarette smoker	57 851 (13.7)	35 164 (9.4)	28 735 (20.0)	21 987 (15.0)	143 737 (13.2)
Ex-smoker	100 780 (23.9)	93 502 (24.9)	46 589 (32.5)	50 380 (34.3)	291 251 (26.8)
Never smoked	262 448 (62.3)	247 397 (65.8)	68 152 (47.5)	74 693 (50.8)	652 690 (60.0)
Total	421 079 (100.0)	376 063 (100.0)	143 476 (100.0)	147 060 (100.0)	1 087 678 (100.0)

a Pearson χ^2^ (6) = 25 000. *P < .*001 [cell values are: count (column %)].

### Relative risks by disability status and pandemic period (unadjusted for survey year)

**Figure 1.**
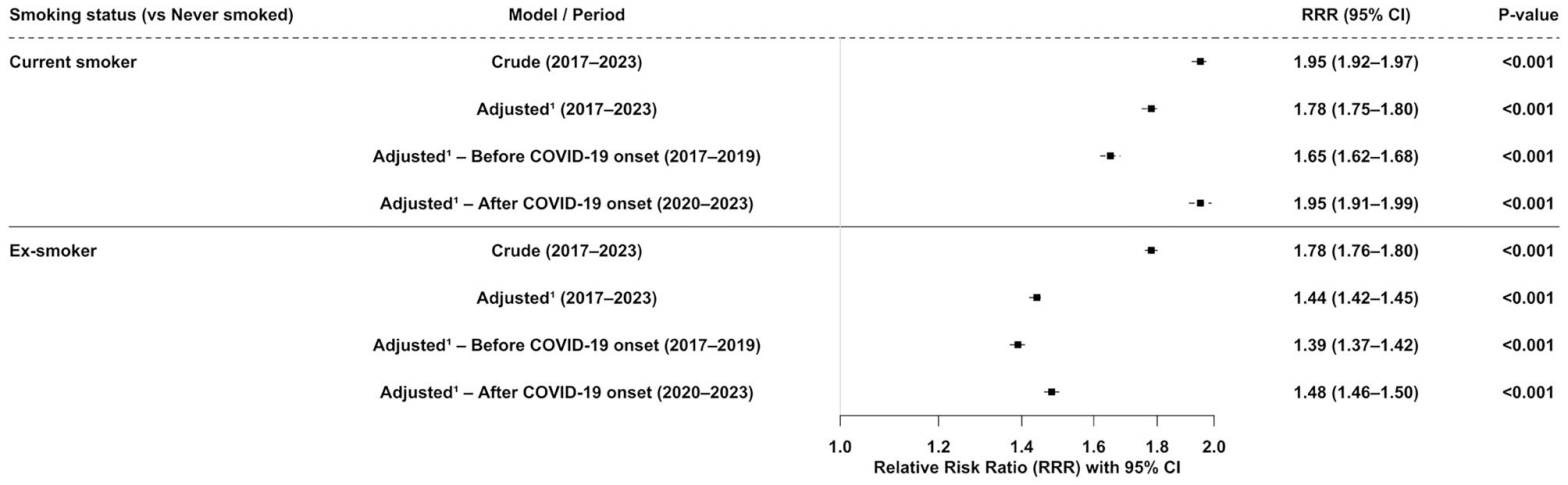
Crude and adjusted relative risk ratios (RRRs) for the association between disability and smoking status, overall and by period relative to COVID-19 onset. ^1^Adjusted for age group, sex, education, ethnicity, marital status, and government office region. Because this model does not adjust for survey year, the estimated differences reflect period contrasts without accounting for underlying time trends.

Figure 1 shows crude and adjusted relative risk ratios (RRRs) for the association between disability and smoking status, overall and by period relative to the onset of the COVID-19 pandemic, using never smoking as the reference category. Across 2017–23, disabled adults in the UK had a 95% higher crude risk of current smoking compared with non-disabled adults (RRR = 1.95, 95% CI: 1.92–1.97, *P < .*001), which remained elevated after adjustment for age, sex, education, ethnicity, marital status, and region (RRR = 1.78, 95% CI: 1.75–1.80, *P < .*001). The adjusted association for current smoking was weaker before the pandemic (RRR = 1.65, 95% CI: 1.62–1.68, *P < .*001) and stronger after (RRR = 1.95, 95% CI: 1.91–1.99, *P < .*001). For ex-smoking, disabled adults had a 78% higher crude risk and a 44% higher adjusted risk compared with non-disabled adults. The adjusted association was slightly weaker before the pandemic (RRR = 1.39, 95% CI: 1.37–1.42, *P < .*001) and slightly stronger after (RRR = 1.48, 95% CI: 1.46–1.50, *P < .*001). These stratified models do not adjust for survey year, so the observed differences reflect simple period contrasts rather than underlying temporal trends.

### Relative risk ratios from a trend-adjusted model testing pandemic versus pre-existing trend effects

To distinguish true pandemic effects from pre-existing year-to-year trends, we next fitted a trend-adjusted model including both survey year and the post-2020 indicator. This model additionally controlled for age group, sex, highest educational attainment, ethnicity, marital status, and government office region. Disability remained strongly associated with smoking status: disabled adults had significantly higher relative risk ratios of being current smokers (adjusted RRR = 1.76, 95% CI: 1.72–1.79, *P < .*001) and ex-smokers (RRR = 1.41, 95% CI: 1.39–1.44, *P < .*001). Although current smoking declined overall after 2020 (RRR = 0.88, 95% CI: 0.85–0.91, *P < .*001), the disability × pandemic period interaction was not statistically significant (χ^2^(2) = 3.11, *P = .*21), indicating no independent pandemic-related shift in the disability–smoking relationship once temporal trends were accounted for. In contrast, the disability × survey year interaction was significant (χ^2^(2) = 80.78, *P < .*001), driven by a yearly increase in the relative risk ratio of current smoking for disabled adults (RRR = 1.06, 95% CI: 1.05–1.08, *P < .*001), demonstrating a pre-existing trend of a widening disparity. However, as shown in Table 3, this manifested as a modest narrowing of the absolute disparity in current smoking prevalence after accounting for temporal trends.

**Table 3.** Adjusted average marginal effects (percentage-point differences, disabled minus non-disabled) in smoking status from the trend-adjusted model, 2017–23^a^

Smoking status	Gap (disabled and non-disabled), before onset (2017–19)% (95% CI)	Gap (disabled and non-disabled), after onset (2020–23)% (95% CI)	Change in gap % (95 % CI)	*P* value
Current smoker	+5.63 (5.30 to 5.96)	+4.60 (4.25 to 4.96)	−1.03 (−1.63 to −0.42)	.001
Ex-smoker	+4.30 (3.90 to 4.71)	+4.94 (4.50 to 5.37)	+0.64 (−0.11 to 1.38)	.095
Never smoked	−9.93 (−10.38 to −9.48)	−9.54 (−10.02 to −9.06)	+0.39 (−0.44 to 1.21)	.357

a Estimates are from a multinomial logistic regression model adjusted for age group, sex, education, ethnicity, marital status, government office region, and survey year (centred at 2019). Average marginal effects represent absolute percentage-point differences in predicted probabilities between disabled and non-disabled adults. Positive values indicate higher prevalence among disabled adults.

### Absolute differences in smoking status (trend-adjusted model)

Table 3 presents adjusted average marginal effects (AMEs) from the trend-adjusted multinomial logistic regression, showing absolute percentage-point differences in smoking status between disabled and non-disabled adults. All models controlled for age, sex, education, ethnicity, marital status, government office region, and survey year (centred at 2019). The adjusted disability gap in the probability of current smoking was 5.63 percentage points (95% CI: 5.30–5.96) before the pandemic and 4.60 percentage points (95% CI: 4.25–4.96) after its onset, indicating a modest narrowing of the gap once underlying temporal trends were accounted for (difference = −1.03 percentage point, 95% CI: −1.63 to −0.42; *P = .*001). For ex-smoking, the gap increased slightly from 4.30 percentage points (95% CI: 3.90–4.71) to 4.94 percentage points (95% CI: 4.50–5.37), although this change was not statistically significant (*P = .*095). Disabled adults remained substantially less likely to have never smoked, with the gap narrowing marginally from −9.93 percentage points (95% CI: −10.38 to −9.48) before the pandemic to −9.54 percentage points (95% CI: −10.02 to −9.06) after 2020 (*P = .*357).

## Discussion

This large, nationally representative analysis of over one million UK adults found substantial and persistent disparities in smoking status between disabled and non-disabled adults from 2017 to 2023. Disabled adults were significantly more likely to be current or ex-smokers and less likely to have never smoked across all survey years. These patterns persisted after adjusting for key sociodemographic factors, suggesting that disability-related smoking disparities cannot be explained solely by age, sex, education, ethnicity, marital status, or region.

The persistence of the disability–smoking gap during the pandemic may reflect a combination of structural and behavioural mechanisms. Disabled adults faced heightened vulnerability to pandemic-related stressors, including social isolation, reduced income, and disruptions to health and social care [22–24]. Simultaneously, restrictions on service provision and redeployment of public health resources may have reduced access to cessation support, with evidence suggesting that service availability and uptake fell markedly in 2020–21 [25]. Although the joint model indicated no discrete change at the onset of the pandemic, declines were broadly parallel across disability status; the small absolute narrowing reflects continuation of pre-existing trends rather than an independent pandemic effect. These findings align with research showing that disadvantaged populations often experience less benefit from population-wide tobacco control gains, leading to entrenched inequalities over time [26–28].

Higher ex-smoking prevalence among disabled adults, observed before and after the pandemic, likely reflects both higher baseline smoking initiation rates and elevated quit attempts due to health deterioration. However, the persistently high proportion of current smokers underlines the difficulty of sustaining cessation in the context of disability-related barriers, including chronic pain, mental distress, and socioeconomic disadvantage [23, 27, 28]. The persistent large gap in never smoking is of particular concern, as it signals continued high uptake of smoking among disabled people relative to non-disabled peers. Preventing smoking initiation in younger disabled populations, through targeted prevention campaigns, tailored school-based interventions, and inclusive messaging, should be a key public health priority alongside cessation support.

These findings have clear implications for tobacco control policy. National strategies to achieve the UK government’s target of <5% by 2030 will likely fall short if they do not explicitly address the needs of disabled adults. The prevalence of smoking may even be higher than captured in our data, given that national household surveys exclude ‘hidden’ populations with substantially elevated smoking rates, such as those in institutions, or temporary accommodation [29], underlining the need for greater effort. Evidence-based approaches include tailoring cessation interventions to address comorbidities, improving accessibility of services (both physical and digital), integrating smoking cessation into disability support pathways, and training healthcare professionals to deliver sensitive, non-stigmatizing support. Structural interventions, such as tackling poverty and housing insecurity among disabled populations, may indirectly reduce smoking prevalence by addressing underlying determinants. Our results also highlight the need to monitor tobacco use trends by disability status in routine surveillance to ensure progress is equitable.

This study has several strengths, including its use of a large, nationally representative dataset with consistent disability and smoking measures over a 7-year period, and the application of both relative and absolute measures of inequality. However, limitations should be acknowledged. The cross-sectional design precludes causal inference about the impact of the pandemic on individual smoking behaviour. Self-reported smoking status may be subject to recall or social desirability bias, although such misclassification is unlikely to vary systematically by disability. Operational and mode changes in the APS during the pandemic, particularly the shift from face-to-face to telephone data collection, may also have influenced reporting. A further limitation is the substantial missingness in education data, especially in 2022–23, largely due to changes in questionnaire routing and proxy responses. The APS definition of disability follows the Equality Act, which may not capture all relevant functional limitations. Residual confounding from unmeasured variables such as income, mental health status, or access to cessation services is possible. Despite these caveats, the observed trends are consistent, robust across sensitivity analyses, and point to a persistent public health gap that warrants urgent attention.

From 2017 to 2023, disabled adults in the UK were consistently more likely to be current or ex-smokers and less likely to have never smoked compared to non-disabled adults, even after adjusting for sociodemographic factors. Despite a slight narrowing of the smoking gap, these disparities persisted, highlighting the urgent need for targeted tobacco control strategies that address structural, social, and health-related barriers faced by disabled individuals. Without prioritizing disability in national smoke-free plans and routine monitoring, efforts to reduce smoking may widen existing health inequalities.

## Data Availability

Office for National Statistics, Social Survey Division. (2017–23). Annual Population Survey [data collection]. UK Data Service. Series ID 200002. See individual study datasets (e.g. SN 8789 for January–December 2020). https://datacatalogue.ukdataservice.ac.uk/series/series/200002#abstract
